# Modeling Direct and Indirect Action on Cell Survival After Photon Irradiation under Normoxia and Hypoxia

**DOI:** 10.3390/ijms21103471

**Published:** 2020-05-14

**Authors:** Hans Liew, Stewart Mein, Jürgen Debus, Ivana Dokic, Andrea Mairani

**Affiliations:** 1 Clinical Cooperation Unit Radiation Oncology, German Cancer Research Center (DKFZ), 69120 Heidelberg, Germany; h.liew@dkfz-heidelberg.de (H.L.); Juergen.Debus@med.uni-heidelberg.de (J.D.); 2 Division of Molecular and Translational Radiation Oncology, National Center for Tumor Diseases (NCT), Heidelberg University Hospital, 69120 Heidelberg, Germany; s.mein@dkfz-heidelberg.de (S.M.); i.dokic@dkfz-heidelberg.de (I.D.); 3 Heidelberg Institute of Radiation Oncology (HIRO), German Cancer Research Center (DKFZ), 69120 Heidelberg, Germany; 4 German Cancer Consortium (DKTK), 69120 Heidelberg, Germany; 5 Heidelberg Ion-Beam Therapy Center (HIT), 69120 Heidelberg, Germany; 6 Faculty of Physics and Astronomy, Heidelberg University, 69120 Heidelberg, Germany

**Keywords:** ionizing radiation, direct and indirect damage, hypoxia, modeling, DMSO, radicals, radical scavengers

## Abstract

The demand for personalized medicine in radiotherapy has been met by a surge of mechanistic models offering predictions of the biological effect of ionizing radiation under consideration of a growing number of parameters. We present an extension of our existing model of cell survival after photon irradiation to explicitly differentiate between the damage inflicted by the direct and indirect (radicals-mediated) action of ionizing radiation. Within our approach, we assume that the oxygenation status affects the indirect action. The effect of different concentrations of dimethyl sulfoxide (DMSO), an effective radical scavenger, has been simulated at different dose levels in normoxic and hypoxic conditions for various cell lines. Our model is found to accurately predict experimental data available in literature, validating the assumptions made in our approach. The presented extension adds further flexibility to our model and could act as basis for further developments of our model.

## 1. Introduction

Nearly 50% of cancer patients are treated with some form of radiation therapy during the course of the disease [1] with recent trends shifting towards more personalized planning and delivery. Innovative treatments, however, require development, validation and clinical translation of highly detailed and accurate physical and biological models for normal tissue and tumor response, considering various bio-factors based on both measurable quantities, discovered mechanisms and theory [2]. 

In this manuscript, we present an extension of the “UNIfied and VERSatile bio response Engine” (UNIVERSE) biological modeling environment [3], which is progressively extended by mechanistic implementations of biological processes relevant for the ultimate radiation response of cells. In a recent publication [3], the cellular response of hypoxic cells in combination with the administration of radio-sensitizing drugs, such as DNA damage response (DDR) inhibitors was implemented into UNIVERSE [4,5,6,7]. In this work, consequences of direct and indirect (radicals-mediated) damage in both conditions of normoxia and hypoxia are explicitly considered and incorporated into the UNIVERSE framework. In doing so, we obtain an improved understanding and predictability of the effect of hypoxia, which is known to be highly relevant to treatment outcomes in radiation clinics [8,9,10].

It is generally accepted, that the induction of DNA damage by photons can be separated into two pathways: direct actions due to the deposition of radiation energy directly on the DNA molecule and indirect actions, which are mediated by formation of free radicals and their interaction with cellular structures [11,12]. Due to this distinct role of free radicals in radiation induced damage, chemical compounds that act as free radical scavengers are under consideration as potential agents to minimize side effects in radio therapy [13,14] or reduce the probability of carcinogenesis after exposure to ionizing radiation from diagnostic imaging [15]. In particular, dimethyl sulfoxide (DMSO) is an effective scavenger of the OH radical, one of the main mediators of low linear energy transfer (LET) radiation induced cellular damage, and has been widely used to investigate the indirect action of ionizing radiation [12,16,17,18].

The increased radioresistence of cells under absence of free oxygen (oxygen effect) is most commonly explained by the oxygen fixation hypothesis: molecular oxygen can react with radicals produced in the DNA, which directly competes with its chemical restoration by reaction with H+, and thus fixing part of the damage [8,19,20]. However, the results of several studies investigating both cell survival [12,21] and extent of DNA damage [21,22], have suggested that the oxygen effect predominantly affects the indirect action of damage induction, while the direct action is only weakly modified.

In the previous version of UNIVERSE, predictions were based on a general double strand break (DSB) yield ($\alpha_{DSB}$), independent of the underlying mechanism. Now, we implement a cell line independent separation of this yield into a direct fraction and an indirect fraction. Furthermore, we consider a free radical scavenger such as DMSO to reduce solely the yield of the indirect fraction. In the first part of this work, we determined an empirical parametrization of the fraction of the indirect damage quenched by a given DMSO concentration ($f_{DMSO}$), based on data from three different cell lines in the literature. In order to validate the approach, we compared the predicted relative DSB damage yield over a range of DMSO concentrations to measurements from the literature. 

In an earlier publication [3], it was demonstrated, that one can successfully account for the oxygen effect by simply modifying the total DSB yield by a hypoxia reduction factor ($HRF_{DSB}^{O_{2}}$) resembling the classical oxygen enhancement factor (OER) for a given oxygen concentration. Based on the evidence mentioned above we assume that, while the total DSB yield is still being reduced as before, the yield corresponding to the direct pathway of damage induction is unaffected by the oxygen status. This assumption is benchmarked in the second part of this work by applying the previously derived $f_{DMSO}$ to the reduced yield corresponding to the indirect damage induction for two cell lines under hypoxia at three different dose levels over a range of DMSO concentrations. 

## 2. Results

### 2.1. Parametrization of the Effect of DMSO on the Indirect Damage in Our Model

**Figure 1:**
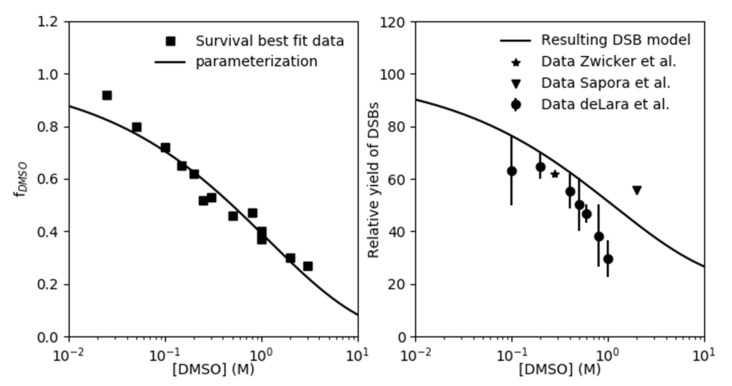
Left panel: $f_{DSMO}$ parameterization as function of DMSO in units of M is depicted with line together with best-fit $f_{DSMO}$ data (squares) obtained by reproducing the cell survival data in normoxia. Right panel: predicted initial normoxic yield of DSBs relative to the yield in absence of DMSO ($R_{DSB}$) calculated using Equation (9) is compared against the experimental data from the literature.

**Figure 2:**
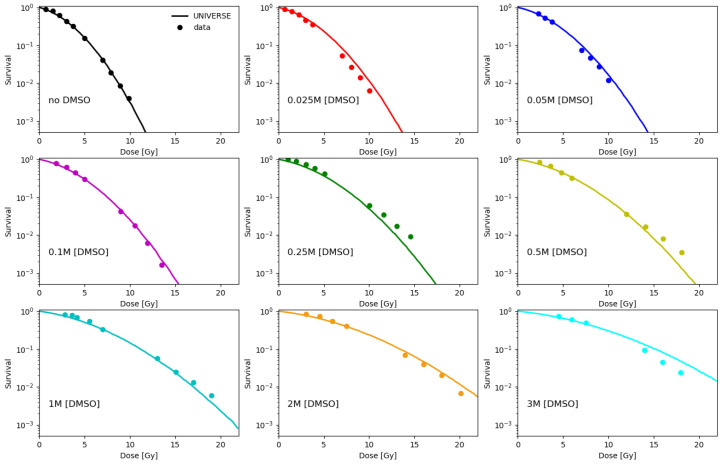
Model predictions (lines) based on the parametrization found in Figure 1 (lines) are plotted together with cell survival data (dots) of V79 cell line irradiated under normoxia without and with different DMSO concentrations ranging from 0.025 M to 3 M, taken from Chapman et al. [16].

**Figure 3:**
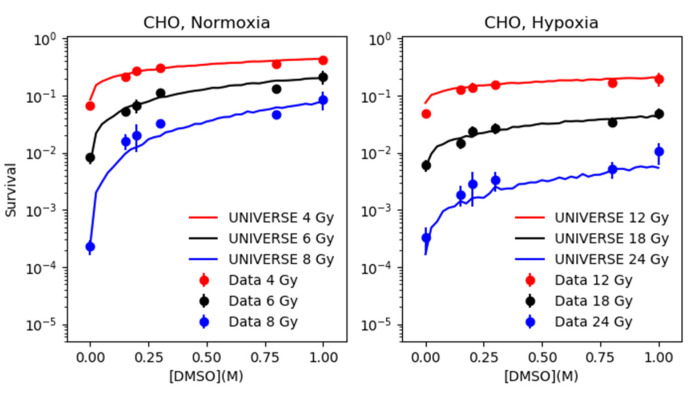
CHO cell survival data (dots with error bars) in normoxia (left panel) and in hypoxia (right panel) as a function of DMSO concentration in units of M for different dose levels as shown in the legends, taken from Hirayama et al. [12], are compared against model predictions (lines).

Fitting the normoxic survival measurements without application of DMSO (data of Hirayama et al. [12] and Chapman et al. [16]), the cell line specific lethality parameters of our model $K_{iDSB}$ and $K_{cDSB}$ were derived for the V79, CHO and AA8 cell-line. These parameters indicate the probability of an isolated (iDSB) and complex DSB (cDSB) leading to the cell’s inactivation (see Materials and Methods). The numerical results are summarized in Table 1. Based on the contribution of indirect action found by Hirayama et al. [12] we assume the fraction of DSB yield based on the direct action ($fr_{DIR}$) to be 20%. Based on this assumption, the fraction of indirect damage quenched by DMSO ($f_{DMSO}$) was tuned for each DMSO concentration measured by Chapman et al. [16] and Hirayama et al. [12] so that our model would reproduce the observed survival trends under normoxia (data in Figure 2 and data in left panel of Figure 3 for CHO cells at 4 Gy under normoxia). Values determined for $f_{DMSO}$ are shown in the left panel of Figure 1 together with a cell-line independent empirical fit of the data. The model predictions shown in Figure 2 were calculated based on this parametrization. As further validation of our approach, we calculated the relative yield of DSBs as the ratio between the DSB without application of DMSO to values of measurements over a range of DMSO concentrations taken from literature (right panel, Figure 1), which have been measured using a variety of techniques [21,23,24]. 

### 2.2. Modeling the Effect of DMSO under Normoxia and Hypoxia

**Figure 4:**
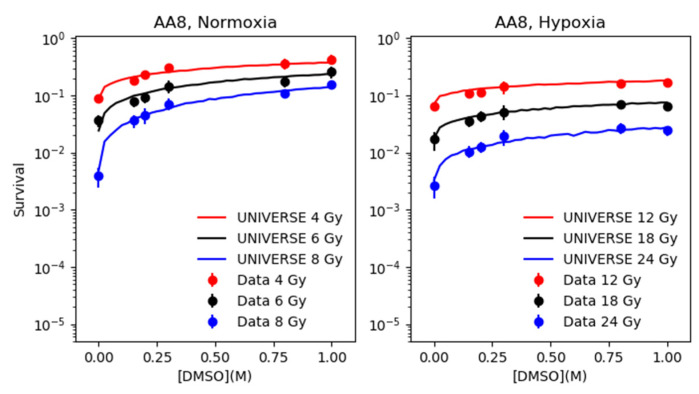
AA8 cell survival data (dots with error bars) in normoxia (left panel) and in hypoxia (right panel) as a function of DMSO concentration in units of M for different dose levels as shown in the legends, taken from Hirayama et al. [12], are compared against model predictions (lines).

Based on the parametrization of $f_{DMSO}$ determined in the previous section, the survival of CHO and AA8 cells were predicted by our model over the range of 0.0 M to 1.0 M of DMSO at three different dose levels and compared to the data taken from Hirayama et al. [12] (left panels of Figure 3 and Figure 4). In order to extend our predictions to the hypoxic case, the $HRF_{DSB}^{O_{2}}$ was tuned to resemble the survival observed without application of DMSO under hypoxia (Supplementary Figure S1). The numerical values found for $HRF_{DSB}^{O_{2}}$ for both cell-lines can be found in Table 1. The derived $HRF_{DSB}^{O_{2}}$ was applied to the total yield of DSB, from which subsequently the fixed yield based on the direct action ($\alpha_{DSB}\cdot fr_{DIR}$) is subtracted to determine the yield based on the indirect action. The reduction determined by the parametrization of $f_{DMSO}$ is applied only to the latter. Finally, the survival is predicted based on the sum of the unchanged yield based on the direct action and the reduced yield based on the indirect action and compared to the data taken from Hirayama et al. [12]. 

## 3. Discussion

The parametrization for the fraction of indirect damage quenched by DMSO ($f_{DMSO}$) carried out in the first part of the analysis (left panel, Figure 1) and its implementation into our model described, in good agreement, the cell survival trends over a wide range of DMSO concentrations at different dose levels for three different cell lines (Figure 2, Figure 3 and Figure 4). In addition, the findings in this work suggest that the rough estimate made for the DSB yield by direct action of ~20% sufficiently described cell survival under the given settings. The selected $f_{DMSO}$ parametrization underestimates the DMSO best-fit scaling value for about 10% at the lowest DMSO concentration. Of course, an improved fitting could be achieved increasing the number of free parameters of $f_{DMSO}$ parametrization; however, in this study we opted for model simplicity (2 free parameters), which itself was capable of replicating the general trends of the $f_{DMSO}$. Furthermore, the $f_{DMSO}$ data point discussed above corresponds to cell survival measurements at 0.025 M, which are still satisfactorily estimated by our model (upper middle panel of Figure 2). The prediction for the ratio of DSB measured with and without DMSO (right panel, Figure 1) generally lies above the values observed in literature [21,23,24]. At low DMSO concentrations (below 0.5 M), the prediction overestimates the measured $R_{DSB}$ by about 10%. At concentrations above 0.6 M, the data of deLara et al. indicate a steeper decline in DSB yields, which they account to unspecified “additional modes of protection” [23,25]. We could not observe this effect in the investigated cell survival data set. The absence of such steep decline could indicate an underlying cell type specific response to irradiation. The work by Kashino et al. [26] implies possible interaction of DMSO with the DSB repair mechanisms, which we possibly could account for by a modification of the lethality parameter of the isolated DSB ($K_{iDSB}$) as we had demonstrated in an earlier publication [3]. On the other hand, Sapora et al. [21] observed significantly higher DSB yields at higher concentrations of DMSO. Based on this, we believe that our predictions are within the range represented by the experimental data.

The extension of our approach introduced here, which includes the effect of hypoxia, results in excellent predictions of the experimental survival data over a range of DMSO concentration at three different dose levels each for two different cell lines (Figure 3 and Figure 4). It shall be emphasized here, that the parametrization of $f_{DMSO}$ is based only on the cell survival data from Figure 2 and the data points of the left panel of Figure 3 (CHO) at 4 Gy without any further adjustments to predict measured cell survival data in hypoxia. The ability of our extension to predict the cell survival under hypoxia with such precision for both cell lines at all three dose levels and over the observed range of DMSO concentrations, suggests that our simplistic assumption, which completely excludes the direct action from the oxygen effect, is valid.

In conclusion, we could demonstrate, that we can introduce a simple mechanistic differentiation of direct and indirect action into our existing model and accurately predict cell survival for different levels of free radicals reduction via DMSO. Not only could we show this for different cell-lines and irradiation dose levels but also under different cell oxygenation states (i.e., normoxia and hypoxia). Ultimately, this adds to the existing versatility of UNIVERSE to consider multiple radio-biologically relevant parameters in its predictions of cell survival, as it can now account for manipulations of the indirect action for a given oxygenation status, e.g., by administered radical scavengers. Furthermore, the assumption that the direct action was unaffected by the oxygenation status, which was here successfully shown for photons, will be considered as a basis for a hypoxia model for the planned heavy-ion extensions of UNIVERSE. 

## 4. Materials and Methods

### 4.1. Experimental Data from Literature

The experimental survival data used to benchmark the extensions of our model were taken from Hirayama et al. [12] and Chapman et al. [16]. The experimental data of the DSB yield as function of [DMSO] were collected from Sapora et al. [21], deLara et al. [23] and Zwicker et al. [24]. 

### 4.2. Modeling Approach

The modeling approach of the basic model has been extensively described and discussed in previous publications [3,27]. However, we must reiterate the key points in an abridged version as folows: for an irradiation with photons we assume the dose deposition to be homogeneous throughout the cell nucleus and a cell line independent DSB yield of $\alpha_{DSB}=5\times 10^{-3}DSB/\left(Mbp\times Gy\right)$ (bp = base pairs), which is constant over the dose range typically applied in the clinic [28,29,30,31]. The expected total number of DSB in the cell nucleus ($‹N_{tDSB}›$) is therefore given by:

(1) $$‹N_{tDSB}›=\alpha_{DSB}\times D\times DNA_{c}$$

where $DNA_{c}$ is the DNA content of a cell in Mbp and *D* the applied dose in units of Gy. In our base model, as in similar models of other groups [32,33,34], so called giant loops, a type of chromatin sub-structure [35,36,37], are believed to be the critical targets in the cell. The total number of giant loops ($N_{gl}$) with a DNA content of $DNA_{gl}$ inside the nucleus is given by:

(2) $$N_{gl}=\frac{DNA_{c}}{DNA_{gl}}$$

As in previous works DNAc and $DNA_{gl}$ were assumed to be 6 Gbp and 2 Mbp, respectively.

The number of total DSB in the nucleus ($N_{tDSB}$) is sampled following a Poisson distribution by a Monte Carlo routine, with the expectation value given by Equation (1). Thereafter, the sampled amount of DSBs is randomly distributed over the giant loops in the nucleus and the number of giant loops with one DSB (isolated DSB; $N_{iDSB}$), or two or more DSBs (complex DSB; $N_{cDSB}$) are scored. The lethality parameters, $K_{iDSB}$ and $K_{cDSB}$, quantify the probabilities of an isolated lesion and a complex lesion leading to the inactivation of the cell, respectively. The overall probability of a cell to survive (*S*) can then be calculated using [33]:

(3) $$S={\left(1-K_{iDSB}\right)}^{N_{iDSB}}\times {\left(1-K_{cDSB}\right)}^{N_{cDSB}}$$

The survival fraction of a cell population after irradiation was determined by the average *S* value determined by the Monte Carlo algorithm. The cell line dependent lethality parameters, $K_{iDSB}$ and $K_{cDSB}$, can be determined by fitting the result of this algorithm to experimental survival data.

In previous works it was shown that in our model a change in oxygenation solely leads to a reduction of the DSB yield, by a hypoxia reduction factor ($HRF_{DSB}^{O_{2}}$) resembling the classical oxygen enhancement factor (OER), while the lethality parameters could be assumed constant [3,27]. The reduced DSB yield, $\alpha_{DSB}^{O_{2}}$, is given by:

(4) $$\alpha_{DSB}^{O_{2}}=\frac{\alpha_{DSB}}{HRF_{DSB}^{O_{2}}}$$

where $\alpha_{DSB}$ is the rate under normoxia.

Based on Equation (1), the alteration of $\alpha_{DSB}$ leads to a change of $N_{tDSB}$ to $N_{tDSB}^{O_{2}}$. This again leads to alterations of $N_{iDSB}$ and $N_{cDSB}$ to $N_{iDSB}^{O_{2}}$ and $N_{cDSB}^{O_{2}}$, respectively. With lethality parameters invariant under the oxygenation status, the probability of a cell under hypoxic conditions to survive is expressed in our model as:

(5) $$S_{O_{2}}={\left(1-K_{iDSB}\right)}^{N_{iDSB}^{O_{2}}}\times {\left(1-K_{cDSB}\right)}^{N_{cDSB}^{O_{2}}}$$

The $HRF_{DSB}^{O_{2}}$ value for a given oxygenation level can be determined by fitting the model to the hypoxic survival data, while keeping $K_{iDSB}$ and $K_{cDSB}$ constant. However, if either hypoxic or normoxic data are not available, the $HRF_{DSB}^{O_{2}}$ for a given oxygen concentration $\left[O_{2}\right]$ can be estimated using the following formula:

(6) $$HRF_{DSB}^{O_{2}}=\frac{m\times K+\left[O_{2}\right]}{K+\left[O_{2}\right]}$$

where m and K are the maximum value and the turning point of the function, respectively. The parametrization was introduced in a previous work [27], first proposed by Carlson et al. [38] and originally inspired by works of Alper and Howard-Flanders [39]. Our current best fit of available data with this parametrization yields m = 2.94 and K = 0.129% [27].

In our extension of this model for this work, we introduced a distinction of direct and indirect (radical-mediated) DSB induction rate. Based on the work of Hirayama et al. [12], we assumed that the fraction of DSB yield based on direct action ($fr_{DIR}$) is 20% of the total DSB yield. Further, we consider the reduction of the DSB yield through DMSO as an OH radical scavenger, by a given factor $f_{DSMO}$, only to apply to the fraction of DSB yield based on indirect action. Equation (1) can be then rewritten as:

(7) $$‹N_{tDSB}›=fr_{DIR}\times \alpha_{DSB}\times D\times DNA_{c}+\left(1-fr_{DIR}\right)\times \alpha_{DSB}\times D\times DNA_{c}\times f_{DMSO}$$

The $f_{DSMO}$ values in the left panel of Figure 1 were obtained by tuning the predicted cell survival resulting from Equation (7) to reproduce the experimental survival data shown in Figure 2 and the 4 Gy normoxic data of the CHO cells (left panel, Figure 3). Limiting the number of free parameters (<3) for model simplicity, the parametrization used to fit these values was:

(8) $$f_{DMSO}=\exp\left(-a_{DMSO}\times {\left[DMSO\right]}^{b_{DMSO}}\right)$$

where $a_{DMSO}$ = 0.9065 and $b_{DMSO}$ = 0.4172 and [DMSO] is the DMSO concentration in units of M. The function approaches unity and zero for vanishing and for increasing DMSO concentrations, respectively. The predicted initial yield of DSBs in normoxia relative to the yield in absence of DMSO ($R_{DSB}$) is calculated as:

(9) $$R_{DSB}=(fr_{DIR}+\left(1-fr_{DIR}\right)\times f_{DMSO})\times 100$$

Various experimental investigations have implied, that both for cell survival [12,21] and induction of DNA damage itself [21,22], the oxygen effect primarily interferes with the indirect pathway of damage induction, while the direct pathway is only weakly affected. In our model, we assume that the fraction of DSB yield based on the direct action is completely unaffected by the oxygen affect. In the hypoxic case, Equation (7) therefore transforms to:

(10) $$‹N_{tDSB}^{O_{2}}›=fr_{DIR}\times \alpha_{DSB}\times D\times DNA_{c}+\left(\alpha_{DSB}^{O_{2}}-\alpha_{DSB}\times fr_{DIR}\right)\times D\times DNA_{c}\times f_{DMSO}$$

Throughout the calculations, the lethality parameters $K_{iDSB}$ and $K_{cDSB}$ are kept constant and the simulation of the survival fraction is carried out analogously to the procedure described above, only replacing Equation (1) with Equation (7) or Equation (10), for normoxia or hypoxia, respectively.

## Figures and Tables

**Table 1:** Model parameters derived from cell survival data of three cell lines irradiated with different DMSO concentrations and in hypoxic condition when available.

Cell Line	$K_{iDSB}$	$K_{cDSB}$	$HRF_{DSB}^{O_{2}}$	Reference
CHO	5.56 × 10^−3^ ± 1.12 × 10^−3^	7.65 × 10^−1^ ± 0.44 × 10^−1^	2.90	Hirayama et al. 2013 [12]
AA8	14.00 × 10^−3^ ± 1.48 × 10^−3^	9.16 × 10^−1^ ± 0.89 × 10^−1^	2.85	Hirayama et al. 2013 [12]
V79	4.79 × 10^−3^ ± 0.52 × 10^−3^	3.17 × 10^−1^ ± 0.13 × 10^−1^	–	Chapman et al. 1979 [16]

## Supplementary Materials

Supplementary materials can be found at https://www.mdpi.com/1422-0067/21/10/3471/s1.

## Abbreviations

LET	Linear energy transfer
DMSO	Dimethyl sulfoxide
UNIVERSE	UNIfied and VERSatile bio response Engine
DSB	(DNA) Double strand break
iDSB	Isolated DSB
cDSB	Complex DSB
OER	Oxygen enhancement ratio
HRF	Hypoxia reduction factor
CHO	Chinese hamster ovary
