# Supplementary material for: Modeling Direct and Indirect Action on Cell Survival After Photon Irradiation under Normoxia and Hypoxia
Source: Int J Mol Sci. 2020 May 14;21(10):3471. doi: 10.3390/ijms21103471 (PMC7278970; doi:10.3390/ijms21103471)
Supplement: Supplementary file 1 [file ijms-21-03471-s001.pdf]

Supplementary Material

In Supplementary Figure 1, CHO (left panel) and AA8 (right panel) survival data as function of dose in normoxia (red lines and points) has been fitted by our model to obtain K_{iDSB} and K_{cDSB}. Keeping these parameters fixed, for each cell line, the specific $HRF_{DSB}^{O_2}$ for the hypoxic conditions were fitted (black lines and points). The determined numerical values of each parameter can be found in Table 1 of the main text. Our model shows excellent capability in describing the acquired data for both cell data in normoxia and hypoxia.

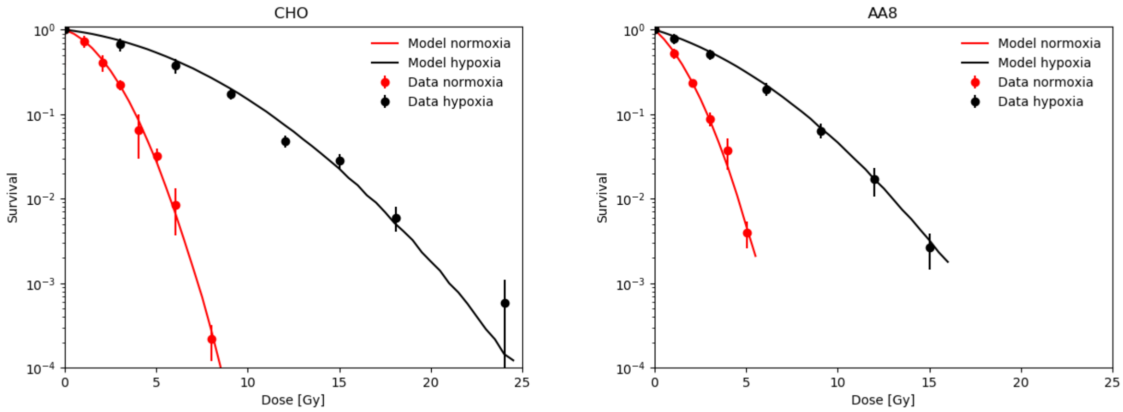

Supplementary Figure 1. Cell survival data of two cell lines (CHO left panel, AA8 right panel) irradiated under normoxia (red) and under extreme hypoxia (black) fitted by the model. The derived $HRF_{DSB}^{O_2}$ values are 2.90 and 2.85, for CHO and AA8, respectively. The experimental data are taken from Hirayama et al. [12].
